# Loop Replacement Enhances the Ancestral Antibacterial Function of a Bifunctional Scorpion Toxin

**DOI:** 10.3390/toxins10060227

**Published:** 2018-06-04

**Authors:** Shangfei Zhang, Bin Gao, Xueli Wang, Shunyi Zhu

**Affiliations:** Group of Peptide Biology and Evolution, State Key Laboratory of Integrated Management of Pest Insects and Rodents, Institute of Zoology, Chinese Academy of Sciences, 1 Beichen West Road, Chaoyang District, Beijing 100101, China; zhangshangfei@ioz.ac.cn (S.Z.); gaob@ioz.ac.cn (B.G.); wangxueli@ioz.ac.cn (X.W.)

**Keywords:** scorpion K^+^ channel toxin, MeuTXKα3, defensin, loop, scaffold

## Abstract

On the basis of the evolutionary relationship between scorpion toxins targeting K^+^ channels (KTxs) and antibacterial defensins (Zhu S., Peigneur S., Gao B., Umetsu Y., Ohki S., Tytgat J. Experimental conversion of a defensin into a neurotoxin: Implications for origin of toxic function. Mol. Biol. Evol. 2014, 31, 546–559), we performed protein engineering experiments to modify a bifunctional KTx (i.e., weak inhibitory activities on both K^+^ channels and bacteria) via substituting its carboxyl loop with the structurally equivalent loop of contemporary defensins. As expected, the engineered peptide (named MeuTXKα3-KFGGI) remarkably improved the antibacterial activity, particularly on some Gram-positive bacteria, including several antibiotic-resistant opportunistic pathogens. Compared with the unmodified toxin, its antibacterial spectrum also enlarged. Our work provides a new method to enhance the antibacterial activity of bifunctional scorpion venom peptides, which might be useful in engineering other proteins with an ancestral activity.

## 1. Introduction

Scorpion venom contains a variety of biologically active peptides for predation and defense, in which peptide toxins affecting Na^+^ and K^+^ channels are the most well-studied components [1,2]. Most of them fold into a common cysteine-stabilized α-helix and β-sheet (CSαβ) structure stabilized by three or four disulfide bridges, a structural motif shared by plant trypsin inhibitors and plant/animal antimicrobial defensins [3,4]. To date, about 250 K^+^ channel toxin (KTx) sequences have been identified, which can be divided into three main groups according to their sequence similarity and phylogenetic relationship: α-, β-, and γ-KTx [5,6,7]. α-KTxs are the largest group of KTXs that usually contain 28–45 residues and bind to several different types of K^+^ channels [8].

Antimicrobial peptides (AMPs) are an important component of the innate immune system in nearly all living organisms. They are active against a diversity of microorganisms, including bacteria, fungi, protozoans, and viruses [9,10]. AMPs are usually small, cationic, and amphipathic molecules with positively charged amino acids and a substantial fraction of hydrophobic residues separated into two domains [11]. They are divided into three main categories: linear peptides, cysteine-containing peptides, and peptides rich in specific amino acids [12]. Defensins with a typical CSαβ structure belong to the second category [3,4].

Using experimental evolution, Zhu et al. have provided convincing evidence in favor of the evolutionary relationship between CSαβ-type antibacterial defensins and α-KTxs [13]. For these two classes of molecules, their carboxyl loop (c-loop) regions connecting the two antiparallel β-strands are involved in interactions with their respective targets. For example, an evolutionarily conserved asparagine in the loop of α-KTxs binds to the channel pore [13,14,15] whereas residues in the loop of defensins bind to bacterial membrane [16,17,18,19].

**Figure 1:**
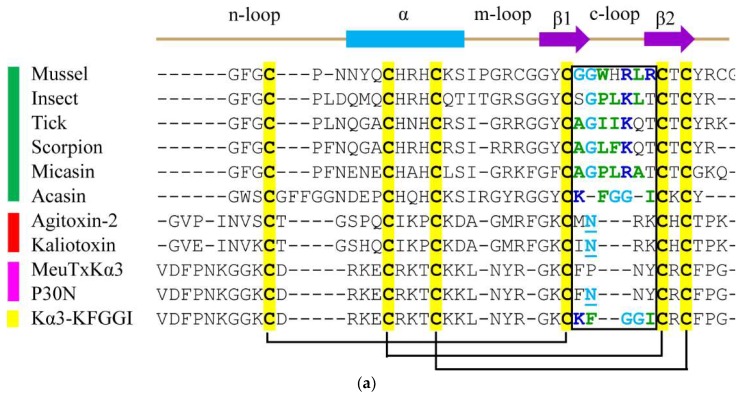

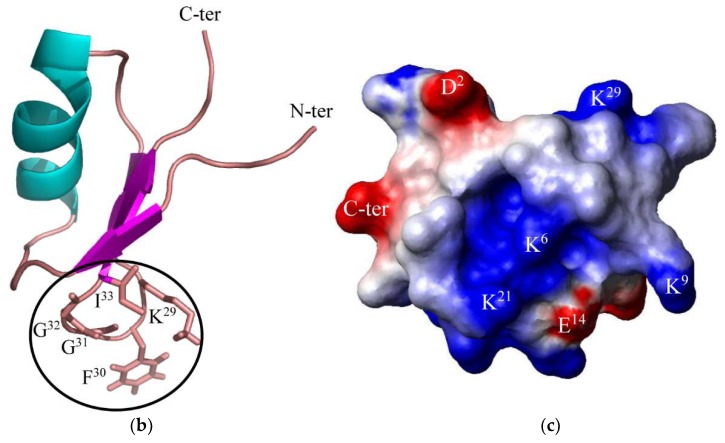
Molecular design of Kα3-KFGGI via loop substitution. (**a**) Multiple sequence alignment of defensins (green bar), scorpion venom-derived K^+^ channel toxins (red bar) and bifunctional scorpion toxins (pink bar), and the designed peptide (yellow bar). Secondary structure elements (α-helix, cylinder; β-strand, arrow) and disulfide bridges are extracted from the NMR structure of micasin (pdb entry 2LR5). Sequence sources: Mussel [23]; Insect [24]; Tick (GenPept No. ABW08118.1); Scorpion [25]; Micasin and Acasin [22]; Agitoxin-2 [26]; Kaliotoxin [27]; MeuTXKα3 and P30N [21]. The c-loop region is boxed, in which residues involved in antibacterial activity are colored according to their chemical properties (blue, basic; green, hydrophobic; cyan, polar) and an Asn essential for K^+^ channel blocking activity is underlined once; (**b**) The computational structure of Kα3-KFGGI. Five residues introduced by loop substitution are indicated; (**c**) Surface potential distribution of Kα3-KFGGI, calculated by MOLMOL, with negative, positive, and neutral charge zones highlighted in red, blue, and white, respectively.

MeuTXKα3 is a weak α-KTx from the scorpion *Mesobuthus eupeus* with some marginal activity against bacteria [20,21]. Given the origin of this class of toxins from defensins [13], this weak antibacterial activity can be considered as an ancestral function. Our previous study has found that a single point mutation, the substitution of proline at position 30 by an asparagine, in its c-loop region, led to enhanced antibacterial activity accompanied by an increase in its toxic function [21], highlighting the functional importance of this loop in determining the peptide’s activity (Figure 1a). We thus proposed a hypothesis that the replacement of the c-loop of MeuTXKα3 by amino acids from the equivalent loop of defensins might create a new peptide with improved antibacterial activity but declined toxicity.

To test this hypothesis, we substituted the c-loop of MeuTXKα3 with a five-residue motif (KFGGI) (Figure 1a). This motif is present in the c-loop of several fungal defensin-like peptides (e.g., Acasin) [22] (Figure 1a). Despite no functional data available for these peptides, the motif reflects common structural characteristics of the functional c-loop region in many defensins from fungi, scorpions, ticks, dragonflies, and mussels (Figure 1a), which include (a) one or two Gly providing the loop flexibility; (b) one or more positively charged residues and several hydrophobic amino acids forming an amphiphilic surface. The engineered peptide, designated as MeuTXKα3-KFGGI (abbreviated as Kα3-KFGGI) (Figure 1b,c), was expressed in *E. coli* and its antibacterial function was assayed. 

## 2. Results

**Figure 2:**
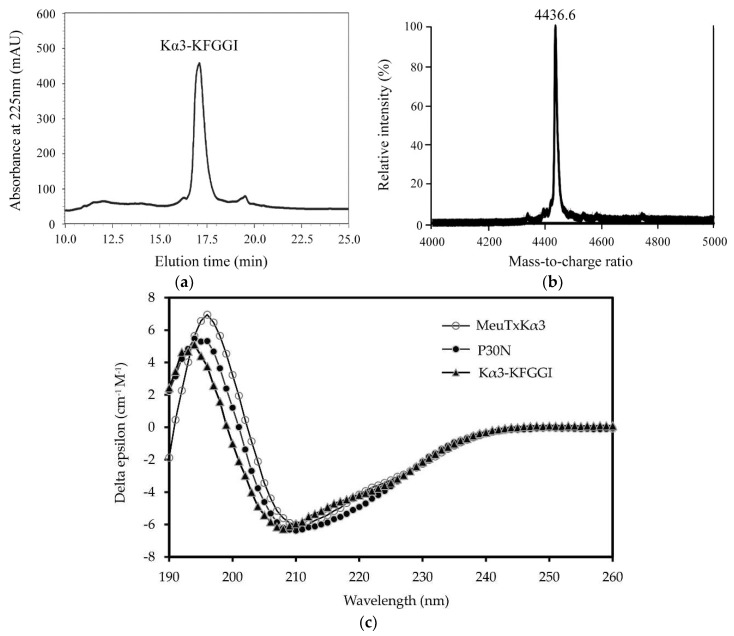
Characterization of recombinant Kα3-KFGGI. (**a**) RP-HPLC isolation of Kα3-KFGGI. The C_18_ column was equilibrated with 0.05% TFA in water (*v*/*v*), and the EK-digested product was eluted from the column with a linear gradient from 0 to 60% acetonitrile in 0.05% TFA within 40 min; (**b**) MALDI-TOF MS determining the molecular mass of Kα3-KFGGI; (**c**) Circular dichroism spectra of Kα3-KFGGI. The spectra were recorded from 190 to 260 nm with a peptide concentration of 0.1 mg/mL in water. MeuTXKα3 and P30N were used as control [21].

The expression and purification of Kα3-KFGGI were carried out according to the method previously reported for MeuTXKα3 [21]. The peptide was expressed in *E. coli* as soluble glutathione S-transferase (GST)-Kα3-KFGGI fusion protein. Following affinity chromatography, the collected fusion proteins were subjected to enterokinase (EK) digestion. The digested product was finally purified by reverse-phase high-performance liquid chromatography (RP-HPLC). Recombinant Kα3-KFGGI was eluted at 17.5 min as a single peak and was detected by MALDI-TOF MS as one single peak at 4436.6 m/z, perfectly matching its theoretical molecular weight of 4436.3 Da (Figure 2a,b). The final expression level was about 100 μg/L bacterial culture.

Using circular dichroism (CD) analysis, we evaluated the secondary structure of the recombinant peptide and made a comparison with two previously reported peptides, MeuTXKα3 and P30N [21]. Our results indicated that the loop substitution overall did not change the structure, as identified by all these three peptides possessing similar CD spectra (Figure 2c). The presence of a negative minimum at 208 nm and a positive maximum at 195–198 nm supports their CSαβ structure.

Subsequently, we compared the bactericidal activity of Kα3-KFGGI with those of MeuTXKα3 and P30N on several species of Gram-positive bacteria using classical inhibition zone assays [22] (Table 1). Of these strains, only three were sensitive to the parent peptide and two of them were sensitive to P30N with some improved activity. All the strains used here, by contrast, were sensitive to Kα3-KFGGI, including several clinical resistant isolates, namely, MRSA, PRSA, and PRSE, and the opportunistic pathogen *Streptococcus salivarius* (Table 1). In particular, Kα3-KFGGI killed penicillin-resistant *Staphylococcus aureus* (P1383) and *Streptococcus salivarius* (CGMCC 1.2498) at extremely low lethal concentrations (0.71–1.34 μM). Except the slightly weaker activity on penicillin-resistant *Staphylococcus aureus* (P1383) than P30N, Kα3-KFGGI displayed a wider spectrum and more significantly enhanced killing activity than the other two peptides (Table 1).

## 3. Discussion

As the first reported bifunctional α-KTx [20,21], the ancestral antibacterial function of MeuTXKα3 provides an opportunity for molecular design to improve this remnant activity [21]. In this work, we successfully replaced its bifunction-associated c-loop region [21] with a structurally equivalent loop from contemporary defensins. The engineered peptide obtained enhanced activity, supporting the feasibility of this strategy in reviving an ancestral peptide activity. Because the whole loop sequence of MeuTXKα3 was substituted by that of the defensin, we conjecture that it may be deficient in K^+^ channel blockade, although this needs to be further investigated. Apart from MeuTXKα3, there exists another group of peptides with dual activities—including cytolytic/antimicrobial activity and the blocking of K^+^ channels in scorpion venom—called β-SPN peptides [28,29,30,31]. Compared with α-KTxs, these peptides evolved an extended N-terminal α-helical domain, which is closely related to their antibacterial activity. Their K^+^ channel toxicity resides in the C-terminal domain that could be modified by our strategy. In addition, a variety of snake venom proteins are evolutionarily related to body proteins with physiological function. Some of them possess the bioactivity of the ancestral proteins [32,33,34,35], which might be suitable candidates for protein engineering.

Our discovery that the substitution of the c-loop of MeuTXKα3 by KFGGI led to enhanced activity indicates that this sequence motif may work as an independent antibacterial unit for protein engineering. In this case, other proteins could be chosen as scaffolds to design new molecules towards antibiotic-resistant bacteria. Because the enhanced peptide retains a similar structure to MeuTXKα3, we believe that this bifunctional peptide may represent a new scaffold for grating of other functionally unrelated motifs. For Kα3-KFGGI, the loop replacement between two unrelated sequences (FPNY vs. KFGGI) with no sequence similarity revealed high tolerance of this peptide to mutations in the loop. We suppose that some conformationally flexible loops responsible for catalysis, binding, or functional switch [36,37,38] are suitable for MeuTXKα3-based protein engineering.

## 4. Conclusions

In summary, our work provides a new strategy for improving the antibacterial activity of a bifunctional scorpion toxin via functional loop replacement, which could be useful in engineering other bifunctional and even multifunctional molecules, such as snake venom proteins. Importantly, as a weak scorpion venom component, MeuTXKα3 is manifested as a new-type protein scaffold for rational design of novel proteins with therapeutic or catalytic activity for clinic and industry application.

## 5. Materials and Methods

### 5.1. Site-Directed Mutagenesis

Inverse PCR, as previously described [39,40], was used to generate the mutant Kα3-KFGGI. The plasmid pGEX-4T-1-MeuTXKα3 [21] previously reported was used as a template, and two primers are listed below: FP: AAATTTGGTGGTATTTGCAGATGTTTTCCAGGATAAGTC; RP: GCATTTTCCTCTGTAATTAAGTTT. Phosphorylation of the 5′-end of the primers was performed by T4 polynucleotide kinase and ATP. Linear PCR products were circularized by T4 DNA ligase after end polishing with Pfu polymerase. Circularized products were transformed into *E. coli* DH5α competent cells, and positive clones were confirmed by DNA sequencing.

### 5.2. Expression, Purification, and Characterization of Recombinant Peptide

The recombinant plasmid of pGEX-4T-1-Kα3-KFGGI was transformed into Rosetta (DE3) chemically competent cells for protein expression. The induction was initiated with 0.1 mM IPTG at an optical density (OD600) of 0.6, and cells were harvested 4 h later by centrifugation. The fusion protein was acquired from the supernatant after sonication, followed by affinity chromatography with Glutathione Sepharose 4B beads. The fusion protein in 1× phosphate-buffered saline (140 mM NaCl, 2.7 mM KCl, 10 mM Na_2_HPO_4_, and 1.8 mM KH_2_PO_4_; pH 7.3) was then digested with EK at 25 °C overnight. The released Kα3-KFGGI proteins were separated from GST by RP-HPLC on a C18 column (Zorbax 300SB-C18, 4.6 × 150 mm, 5 μm) (Agilent Technologies, Santa Clara, CA, USA). The molecular weight of the recombinant molecule was determined by MALDI-TOF mass spectra on ultrafleXtreme MALDI-TOF/TOF Mass Spectrometer (Bruker, USA).

### 5.3. Circular Dichroism Spectroscopy

CD spectra of the peptide were recorded on a Chirascan-plus CD spectrometer (Applied Photophysics, Ltd., Leatherhead, U.K.) at a protein concentration of 0.1 mg/mL dissolved in water. Spectra were measured at 20 °C from 260 to 180 nm with a quartz cell of 1.0 mm thickness. Data were collected at 1 nm intervals with a scan rate of 60 nm/min.

### 5.4. Inhibition Zone Assay

Antibacterial assays were carried out according to the literature [41]. Bacteria were incubated at 37 °C in broth medium until the OD_600_ reached 0.5. In all assays, 10 μL of bacterial culture was mixed in 6 mL of broth medium containing 0.8% agar and were poured into Petri dishes of 9.0 cm diameter. Wells with a diameter of 2 mm were punched into the medium and were filled with 2 μL peptide with different concentrations. Bacteria were incubated at 37 °C overnight, and then zones of inhibition were measured. The lethal concentration (C_L_) was calculated from a plot of d^2^ against log n, where d is the diameter (cm), and n is the amount of the sample applied in the well (nmol). The plot is linear, and thus C_L_ could be calculated from the slope (k) and the intercept (m) of this plot. The formula used here is C_L_ = 2.93/(ak10^m/k^), where a is the thickness of the bacterial plate and C_L_ is in μM [41]. 

### 5.5. Structure Modeling

The model structure of Kα3-KFGGI was built according to the previously described method for MeuTXKα3 [21]. In brief, the solution structure of BmTx3B (PDB entry: 1M2S) was selected as template for comparative modeling with SWISS-MODEL, a fully automated protein-structure homology-modeling server (http://swissmodel.expasy.org), and the model was evaluated by Verify 3D [42]. The modeling process was conducted in the project mode of this server under default parameters. Then the generated model was energy minimized by SPDBV v4.0.1 [43].

## Figures and Tables

**Table 1:** Comparison of lethal concentration (C_L_, μM) of MeuTxKα3, P30N, and Kα3-KFGGI on different bacterial species.

Species	MeuTxKα3	P30N	Kα3-KFGGI
Methicillin-resistant *Staphylococcus aureus* (MRSA), P1374	N.A. ^1^	N.A.	3.69
Penicillin-resistant *Staphylococcus aureus* (PRSA), P1383	5.39	0.87	1.34
Penicillin-resistant *Staphylococcus epidermidis* (PRSE), P1389	N.A.	N.A.	5.35
*Staphylococcus warneri*, CGMCC 1.2824	N.A.	N.A.	5.39
*Streptococcus mutans*, CGMCC 1.2499	33.80	24.06	8.84
*Streptococcus salivarius*, CGMCC 1.2498	N.A.	N.A.	0.71
*Streptococcus sanguinis*, CGMCC 1.2497	3.72	N.A.	2.14

^1^ N.A.: no activity, indicating that no inhibition zone was observed at 1.0 nmol peptide each well.
